# First Insights into the Completely Annotated Genome Sequence of *Bacillus licheniformis* Strain 9945A

**DOI:** 10.1128/genomeA.00525-13

**Published:** 2013-08-01

**Authors:** Michael Rachinger, Sonja Volland, Friedhelm Meinhardt, Rolf Daniel, Heiko Liesegang

**Affiliations:** Georg-August University Göttingen, Institute of Microbiology and Genetics, Department of Genomic and Applied Microbiology & Göttingen Genomics Laboratory, Göttingen, Germanya; Westfälische Wilhelms-Universität Münster, Institut für Molekulare Mikrobiologie und Biotechnologie, Münster, Germanyb

## Abstract

Strains of the species *Bacillus licheniformis* are widely used in biotechnology for the production of enzymes and antibiotics (M. Schallmey, A. Singh, and O. P. Ward, Can. J. Microbiol. 50:1–17, 2004). However, research and application of *B. licheniformis* strains are adversely affected by poor genetic accessibility. Thus, for a closer inspection of natural competence in *B. licheniformis*, the genome of strain 9945A, of which derivatives are known to be naturally competent (C. B. Thorne and H. B. Stull, J. Bacteriol. 91:1012–1020, 1966), was completely sequenced and manually annotated.

## GENOME ANNOUNCEMENT

*Bacillus licheniformis* strain 9945A, a spore-forming soil bacterium with a saprophytic lifestyle, is of biotechnological interest due to its capability to produce polyglutamic acid (1, 2) and the known ability of auxotrophic mutants to exhibit natural competence (3, 4), particularly because the *B. licheniformis* type strain DSM13 displays only faint natural competence (5). For whole-genome sequencing, the wild-type strain was grown in mineral medium (6), and chromosomal DNA was isolated employing the Masterpure DNA purification kit (Epicenter, Madison, WI). The 454 shotgun library was constructed using the GS DNA library preparation kit (Roche 454, Branford, CT) and sequenced with a genome sequencer FLX system (Roche 454). In total, 313,600 reads with 74,174,233 bases were generated and assembled using the Newbler assembly software v1.1 (Roche 454), resulting in 70 contigs >0.5 kb, with 17-fold coverage. Orientation and contig order were determined by using an end-sequenced fosmid library (EpiFos CopyControl fosmid library production kit; Epicenter) and by comparing with the genome of the type strain *B. licheniformis* DSM13 (6, 7). Gap closure was done by PCR-based techniques using the PCR extender kit (5 Prime, Hamburg, Germany), subcloning, and transposon bombing (EZ-Tn5 <TET-1> insertion kit; Epicenter). The sequence was polished to a minimal quality score of Phred 45 (Gap4 software, Staden package [8]). Gene prediction was performed with YACOP (9) and manually curated using the Artemis software (10), and tRNAs and rRNAs were identified by tRNAscan SE (11) and RNAmmer (12), respectively. The functional annotation was performed using the Ergo software (13), including comparison to related organisms, and the Swiss-Prot, NCBI (nr), and InterPro databases (InterPro Scan v4.7, release 24.0/25.0). The final annotation was checked by comparison to the *B. licheniformis* DSM13 reannotation (AE017333) and, when necessary, by additional BLAST searches (Swiss-Prot and InterPro [InterPro Scan v4.8, release 42.0]).

The complete genome sequence of *B. licheniformis* 9945A consists of 4,376,305 bp, organized in one circular chromosome with an average GC content of 45.9%. A total of 4,359 genes have been identified, including 72 tRNAs, 7 rRNA clusters (21 genes), and 40 pseudogenes. For 80% of the 4,226 protein-coding genes (3,378), a function or putative function could be assigned. The *B. licheniformis* 9945A genome is similar to the type strain DSM13 genome with respect to GC content and the number of rRNA and tRNA genes but contains an additional 153 kb. A striking metabolic difference of 9945A and DSM13 concerns the apparent ability of the former to use urea as a nitrogen source (BaLi_c20330 to BaLi_c20410) (14).

Comparative genome analysis revealed that for all genes known to be instrumental in developing natural competence in the closely related Gram-positive model organism *Bacillus subtilis*, orthologs exist in *B. licheniformis* 9945A. Despite such findings, the 9945A wild-type strain displays only poor natural competence compared to the auxotrophic mutants generated and investigated by Thorne and Stull in 1966 (3). Thus, further functional investigations of competence system compounds are necessary and in progress (M. Jakobs, K. Hoffmann, A. Grabke, S. Neuber, M. Liesegang, S. Volland, and F. Meinhardt, submitted for publication).

### Nucleotide sequence accession number. 

The whole-genome sequence of *Bacillus licheniformis* 9945A was deposited in GenBank under accession number CP005965.

## References

[1] Thorne CB Gomez CG Noyes HE Housewright RD 1954 Production of glutamyl polypeptide by Bacillus subtilis. J. Bacteriol. 68:307–315 13201526 10.1128/jb.68.3.307-315.1954 PMC357391

[2] Schallmey M Singh A Ward OP 2004 Developments in the use of bacillus species for industrial production. Can. J. Microbiol. 50:1–17 15052317 10.1139/w03-076

[3] Thorne CB Stull HB 1966 Factors affecting transformation of Bacillus licheniformis. J. Bacteriol. 91:1012–1020 5929742 10.1128/jb.91.3.1012-1020.1966 PMC315992

[4] Gwinn DD Thorne CB 1964 Transformation of Bacillus licheniformis. J. Bacteriol. 87:519–526 14127566 10.1128/jb.87.3.519-526.1964 PMC277048

[5] Hoffmann K Wollherr A Larsen M Rachinger M Liesegang H Ehrenreich A Meinhardt F 2010 Facilitation of direct conditional knockout of essential genes in Bacillus licheniformis DSM13 by comparative genetic analysis and manipulation of genetic competence. Appl. Environ. Microbiol. 76:5046–5057 20543043 10.1128/AEM.00660-10 PMC2916460

[6] Veith B Herzberg C Steckel S Feesche J Maurer KH Ehrenreich P Bäumer S Henne A Liesegang H Merkl R Ehrenreich A Gottschalk G 2004 The complete genome sequence of Bacillus licheniformis DSM13, an organism with great industrial potential. J. Mol. Microbiol. Biotechnol. 7:204–211 15383718 10.1159/000079829

[7] Rey MW Ramaiya P Nelson BA Brody-Karpin SD Zaretsky EJ Tang M Lopez de Leon A Xiang H Gusti V Clausen IG Olsen PB Rasmussen MD Andersen JT Jørgensen PL Larsen TS Sorokin A Bolotin A Lapidus A Galleron N Ehrlich SD Berka RM 2004 Complete genome sequence of the industrial bacterium Bacillus licheniformis and comparisons with closely related bacillus species. Genome Biol. 5:R77 15461803 10.1186/gb-2004-5-10-r77 PMC545597

[8] Staden R Beal KF Bonfield JK 2000 The Staden package, 1998. Methods Mol. Biol. 132:115–130 10547834 10.1385/1-59259-192-2:115

[9] Tech M Merkl R 2003 YACOP: enhanced gene prediction obtained by a combination of existing methods. In Silico Biol. 3:441–451 14965344

[10] Rutherford K Parkhill J Crook J Horsnell T Rice P Rajandream MA Barrell B 2000 Artemis: sequence visualization and annotation. Bioinformatics 16:944–945 11120685 10.1093/bioinformatics/16.10.944

[11] Lowe TM Eddy SR 1997 tRNAscan-SE: a program for improved detection of transfer RNA genes in genomic sequence. Nucleic Acids Res. 25:955–964 9023104 10.1093/nar/25.5.955 PMC146525

[12] Lagesen K Hallin P Rødland EA Staerfeldt HH Rognes T Ussery DW 2007 RNAmmer: consistent and rapid annotation of ribosomal RNA genes. Nucleic Acids Res. 35:3100–3108 17452365 10.1093/nar/gkm160 PMC1888812

[13] Overbeek R Larsen N Walunas T D’Souza M Pusch G Selkov E Liolios K Joukov V Kaznadzey D Anderson I Bhattacharyya A Burd H Gardner W Hanke P Kapatral V Mikhailova N Vasieva O Osterman A Vonstein V Fonstein M Ivanova N Kyrpides N 2003 The Ergo genome analysis and discovery system. Nucleic Acids Res. 31:164–171 12519973 10.1093/nar/gkg148 PMC165577

[14] Rachinger M 2010 Stammdesign in *B. licheniformis*. Ph.D. thesis. Georg-August University, Göttingen, Germany http://hdl.handle.net/11858/00-1735-0000-0006-ADB5-5

